# Target areas to reduce the burden of maternal death due to obstetric hemorrhage in Ethiopia

**DOI:** 10.1371/journal.pone.0274866

**Published:** 2022-09-29

**Authors:** Neamin Tesfay, Rozina Tariku, Alemu Zenebe, Haymanot Firde, Fitsum Woldeyohannes

**Affiliations:** 1 Center of Public Health Emergency Management, Ethiopian Public Health Institutes, Addis Ababa, Ethiopia; 2 Health Financing Program, Clinton Health Access Initiative, Addis Ababa, Ethiopia; Wachemo University, ETHIOPIA

## Abstract

**Background:**

Obstetric hemorrhage is defined as active bleeding of more than 500 ml in vaginal delivery or 1000ml following cesarean delivery. It is the leading cause of maternal death, which contributes to up to 50% of maternal deaths in Ethiopia. This study aims to assess the relationships between adverse maternal health exposure (personal and medical factors) and delay in health care (hesitancy in opting to seek care, lag in reaching a health facility, and wait in receiving health care at the facility) and adverse outcomes of obstetric hemorrhage among reviewed maternal deaths in Ethiopia.

**Methods:**

This study utilizes 4530 reported maternal death surveillance data obtained from Ethiopian maternal death surveillance and response (MDSR) system between 2013 to 2020. Latent class analysis was applied to identify underlying patterns of adverse maternal health exposures. Furthermore, the associations between latent classes and adverse outcomes of obstetric hemorrhage were analyzed using multilevel logistics regression model adjusted for clustering within reporting provinces.

**Results:**

Nearly 56% of the reviewed maternal deaths were due to the adverse outcome of obstetric hemorrhage, among which nearly 75% died during the postpartum period. The study identified six separate sub-groups of women based on their vulnerability to adverse maternal health conditions. The six subgroups identified by this study are 1) women who travelled for a long duration to reach a health care provider, 2) those who had no access to a health facility (HF) within a 5Km radius, 3) those who failed to decide to go to a health facility: 4) those with multiparity,5) those who were injured during delivery with history of coagulopathy, and 6) those who got injured during delivery and failed to decide to go to a health facility. Women in the class of grand multipara have demonstrated the highest risk of death due to the adverse outcomes of obstetric hemorrhage (β = 1.54, SE = 0.09, p<0.0001).

**Conclusions:**

The study has attempted to identify women that are at a higher risk for the adverse outcomes of obstetric hemorrhage. Henceforth, targeted intervention should be taken on women of reproductive age group, and those identified as at a higher risk, to reduce the high rate of maternal death due to obstetric hemorrhage.

## Introduction

A maternal death refers to any death of women of reproductive age, or death within 42 days after the termination of pregnancy, regardless of the duration and site of pregnancy. For a death to be referred to as maternal death, it must be caused or aggravated by the pregnancy or its management, death from accidental or incidental cause is not considered as maternal death [1, 2]. The maternal mortality rate is the commonly used standard to measure the level of maternal death; it is also one of the best indicators to assess the overall socio-economic status of a country [3, 4].

Ethiopia is among the countries that record one of the highest maternal mortality rates (MMR) in Sub-Saharan Africa [5], estimated at 412 deaths per 100,000 live births (LBs), which is a significant reduction compared to the 871 in 2000 [6, 7]. However, although there is a significant reduction in maternal death, Ethiopia was unable to achieve the Millennium Development Goals (MDGs) on maternal health, which had set a target to reduce MMR to 290 per 100000 LBs [8, 9].

Globally, following the termination of MDGs, a new target was set under the Sustainable Development Goal (SDGs), which has the primary objective of reducing the global maternal mortality rate to below 70 deaths per 100,000 LBs, with no country having a maternal mortality ratio of more than twice the global average [10]. By owning the SDG agenda, Ethiopia is set to implement a 20-year ambitious plan that is targeted to reduce MMR to 45.5/10^5^LBs by2035 [11]. The World health organization (WHO) suggests the establishment of a maternal death surveillance and response (MDSR) system in countries where there is a weak vital registration system to monitor the progress of achievements towards the stated goal [12].

The Federal Ministry of Health of Ethiopia launched the national MDSR system in May 2013 to enhance the service quality of maternal health care, particularly during pregnancy, childbirth, and postpartum periods [13, 14]. Currently, the system is actively running throughout all regions of Ethiopia with varying levels of performance. According to the Ethiopian MDSR annual report, obstetric hemorrhage was identified as the leading cause of maternal death for consecutive years since the beginning of the system implementation [15].

Obstetric hemorrhage is defined as active bleeding of more than 500 ml in vaginal delivery or 1000ml following cesarean delivery [16]. However, recently a panel of experts in the field introduced a new set of diagnostic criteria to identify women who are at a higher risk of facing an adverse outcome of obstetric hemorrhage. Accordingly, active bleeding of more than 1000 ml within the first day (24 hours) after birth that fails to stop with the use of initial measures such as uterine massage and uterotonic agents is set to be at higher risk of facing adverse outcomes [17]. Obstetric hemorrhage is an umbrella term used in referring to the timing of maternal bleeding starting from the fetus viability (after pregnancy of 7 months) to the delivery of the fetus and extending up to 42 days after delivery of the fetus, which ultimately results in significant peripartum blood loss [18]. The quality of health care, which is defined by the availability of trained personnel, essential maternal health commodities, and the physical infrastructure can significantly reduce the risk of an adverse obstetric hemorrhage [19, 20]. However, in low-resource settings like Ethiopia, in addition to the health care provider-related factors, delay in deciding to seek care (sociocultural factor) and delay in reaching a healthcare facility (availability and cost of transportation and road condition) could influence the adverse outcomes of obstetric hemorrhage [21, 22]. Furthermore, personal factors (older age and higher parity) and medical factors (uterine atony, coagulopathy, and trauma) are believed to have a connection with an adverse outcome of obstetric hemorrhage [23].

In Ethiopia, since 2000 the contribution of obstetric hemorrhage to maternal death has increased gradually, from around 12% to 22% of the total reported deaths. Before 2000, the top causes of maternal deaths were abortion-related complications and obstructed labor/uterine rupture [24, 25]. A study on trends and causes of maternal death in Ethiopia, based on data from 1990 to 2013, revealed that hemorrhage contributed to 12.2% of total death with a slight increment over time [26]. Other evidence has also pointed out that hemorrhage is becoming the leading cause of maternal mortality, followed by hypertensive disorders of pregnancy. In sharp contrast to this, the share of obstructed labor and abortion has declined over time [27]. Overall, the above-stated studies indicate the presence of a paradigm shift in Ethiopia’s primary cause of maternal death. This was evidenced by the Ethiopian ministry of health’s annual report for 2019, which clearly showed that obstetric hemorrhage has taken the lion’s share in contributing to maternal death [28].

In Ethiopia, there is a lack of ample evidence and literature on the possible risk factors for the adverse outcome of obstetric hemorrhage. Thus, using nationally reviewed maternal death data, this study aims to achieve two objectives 1) to detect the pattern of vulnerability to four domains of exposure relate to maternal health adversities (personal and medical factors, hesitancy in deciding to seek care, lag in reaching a health facility, and wait in receiving health care at the facility) and 2) to test the association between these group and obstetric hemorrhage to produce shreds of evidence that could help in making a policy recommendation for the improvement of maternal health.

## Materials and methods

### Study design

A secondary data review was applied to maternal death surveillance data, from 2013 to 2020.

### Data source

The study used MDSR data collected from different health facilities in Ethiopia. The data collection process goes through notification of maternal death with a review of each death by a designated committee within the reporting health facility. The data was extracted using Facility-Based Abstraction Format (FBAF) and Verbal Autopsy (VA). The established MDSR committee reviews the extracted data to designate the main cause of death and identify contributing factors. Finally, the designated committee prepares a report of each reviewed death using the maternal death reporting format (MDRF). The data is then sent to the national data hub [29].

### Study setting and participants

The total population of Ethiopia is estimated to be 114 million in 2020 [30, 31]. There are nine regional states in the country (namely, Tigray, Afar, Amhara, Oromia, Somali, Benishangul, SNNPR (south nation nationalities people’s region), Gambella, and Harari) and two cities’ administrations (Addis Ababa and Dire Dawa) [32]. A total of 4530 maternal deaths were assessed within the last seven years (2013_to 2020) in Ethiopia’s regions. All reviewed maternal deaths were reported through the MDRF and had detailed and comprehensive information about the deceased women. MDRF obtains information on the reporting facilities, deceased women’s medical and personal information (including antenatal care (ANC) and cause of death).

### Measures and study variables

#### Medical factor

*Older age*. In the study, women who are 35 years old and above were considered as older-aged women [33, 34]. The variable was categorized into two by assigning Yes and No according to the criteria.

*Grand multipara*. A woman with ≥5 parity was considered a grand multipara [35]. The variable has two levels with Yes or No option.

*Being anemic*. A hemoglobin level < 11 g/dl was used as a cutoff point to declare anemia during pregnancy [36]. The variable was dichotomous, with two-level responses assigned with Yes and No options.

*Uterine atony*. It is a condition in which the myometrial fails to constrict blood vessels once the placenta is delivered [37]. Women with detected uterine over destination (multiple pregnancy and Polyhydramnios), uterine muscle fatigue (prolonged labor), uterine infection (prolonged spontaneous rupture of membranes), and placenta previa were eligible for this category. Moreover, women treated with a uterine relaxing agent (anesthetic drugs and nifedipine) were considered under this category [38]. Women identified with one of the diagnoses mentioned above were coded as ‘Yes’, and if not coded with ‘No’.

*Coagulopathy*. It represents an imbalance between the clotting and fibrinolytic systems. Women identified with one of the diagnoses, with acute fatty liver of pregnancy, pulmonary embolism amniotic fluid embolism, Hemolysis Elevated Liver enzymes, and Low Platelets (HELLP) syndrome, deep vein thrombosis, severe preeclampsia, and pre-existing clotting abnormality were included under this category [38, 39]. Response options for coagulopathy were also codified as “Yes” and”No” per the criteria above.

*Injured during delivery*. It may occur with both vaginal and cesarean deliveries. It comprises cervical /vaginal tear (Precipitous delivery, instrumental delivery, and operative delivery) and uterine rupture [39, 40]. Women, who aligned with one of the stated criteria, were coded as ‘Yes’ whereas, if not fit the criteria code with ‘No’.

### Conceptualization of the three-delay model

The model was adopted and contextualized from Thaddeus and Maine framework, and it is mainly used to evaluate the environments and physical settings during the time of maternal death [41]. These are 1) delays due to hesitancy in opting to seek care 2) delays due to lags in reaching the health facility 3) delays due to waits in receiving health care at the healthcare facility. Yes/No questions were employed to assess the three delay models.

Type one delays (delays due to hesitancy in opting to seek care) are influenced by the factors involved in decision-making: sociocultural factors; financial and opportunity costs.

Type two delays (delays due to lags in reaching the health facility) are related to factors such as travel time or distance to the nearest healthcare facility, the road conditions, and availability and cost of transportation.

Type three delays (delays due to waits in receiving health care at the healthcare facility) include factors affecting the speed with which effective care is provided once a woman reaches a health facility (HF), shortages of supplies, equipment, and trained personnel, the competence of available personnel, and quality of care (Table 1).

**Table 1:** General framework used to measure and classify different contributing factors among reviewed maternal death in Ethiopia, 2020.

Delay 1 –due to hesitancy in opting to seek care	Delay 2 –due to lags in reaching a health facility	Delay 3 –due to waits in receiving health care service at the facility
Family poverty (family has insufficient money)	lack of healthcare facilities in the surrounding. (Consumes more than an hour to reach healthcare facility)	Long travel time from a HF to HF፟ (takes more than one hour during referral due to an Inadequate referral system)
Failed to decide to go to HF	Extended travel time from home to a healthcare facility (takes more than an hour)	Scarcity of essential equipment and supplies
Visited a traditional healer or traditional birth attendant first (traditional practice)	Poor road condition (lack of a road)	Waiting for a longer duration before receiving treatment (more than 30 min from the time of arrival to the time of being assessed or receiving treatment)
Having poor knowledge of obstetric complications	Lack of money for transport (cost of transportation)	Mistaken during an assessment, diagnosis, and treatment
The nearest healthcare facility is more than 5 km away	Lack of transportation	

### Data analysis

A Latent Class Analysis (LCA) is a statistical approach usually adopted to identify different subgroups within a population based on certain characteristics [42]. Accordingly, in an effort to identify subgroups of mothers that are at increased risk of adverse outcomes of obstetrics hemorrhage and identify patterns of possible risk factors the analysis approach was utilized. The LCA was carried out within observed indicators under four domains (medical factors, delay due to hesitancy in opting to seek care, delay due to lags in reaching a health facility, and delay due to waits in receiving health care services at the health facility). The LCA was performed in R studio, Version 4.1.2 using the poLCA package [42]. The models were compared based on entropy and the Bayesian Information Criterion (BIC) [43]. Better fitting models, which have higher entropy values and lower BIC values, indicating a superior precision in assigning latent class membership were selected as shown in Table 2.

**Table 2:** Model goodness of fit parameter for the six different class models among reviewed maternal death in Ethiopia, 2020.

Class	LL^a^	BIC	AIC ^b^	Npar ^c^	*Entropy*
**2**	-38992.34	78329.84	78066.68	41	0.72
**3**	-38233.02	76987.99	76590.04	62	0.74
**4**	-38026.46	76751.66	76218.93	83	0.76
**5**	-37869.43	76614.38	75946.86	104	0.79
**6**	-37742.18	76536.66	75734.35	125	0.82

^a^ LL = Log-likelihood

^b^AIC = Akaike Information criterion

^c^Npar = Number of parameters estimated

Finally, a mixed-effects regression model was fitted to examine possible associations between obstetric hemorrhage and latent classes using Stata version 17.0. A random intercept model was run by adjusting for clustering within the province and accounting for possible within-cluster correlation [44]. The final regression model was built by controlling for socio-demographic characteristics (region and residence), level of care, place of death, and history of ANC follow-up.

### Ethical clearance

We used secondary data obtained from the Ethiopian Public Health Institutes (EPHI) with no personal identifier information of the participants. The EPHI Review Board and Public Health Emergency Management Unit approved the research proposal with Ref. No. EPHI 6_5/437

## Result

### Background characteristics of the reporting health facilities

Among the reviewed maternal deaths, 55.5% occurred due to obstetric hemorrhage More than half (57.3%) of the deaths were reported by primary-level health care providers. More than half (58.7%) of the reviewed maternal deaths were reported from Amhara and Oromia regions. Moreover, nearly half (46.9%) of the deaths occurred in 2016 and 2017 (Table 3).

**Table 3:** Selected background characteristics of maternal deaths reviewed from facilities in Ethiopia, 2020 (N = 4530).

Variable	Deceased by obstetric hemorrhage		Total
	No (%), N = 2017	Yes (%), N = 2513	
**Level of care**			
Primary health care unit	997 (38.29)	1,607 (61.71)	2604
Secondary level of care	458 (48.36)	489 (51.64)	947
Tertiary level of care	562 (57.41)	417 (42.59)	979
**Facility ownership**			
NGO	5 (41.67)	7 (58.33)	12
Private	24 (61.54)	15 (38.46)	39
Government	1,988 (44.38)	2,491 (55.62)	4479
**Reporting region**			
Addis Ababa	167 (60.95)	107 (39.05)	274
Afar	53 (67.95)	25 (32.05)	78
Benishangul Gumuz	43 (55.13)	35 (44.87)	78
Amhara	490 (39.20)	760 (60.80)	1250
Gambella	11 (34.38)	21 (65.62)	32
Dire Dawa	99 (60.00)	66 (40.00)	165
Harari	42 (48.28)	45 (51.72)	87
Oromia	576 (40.88)	833 (59.12)	1409
SNNPR	242 (43.06)	320 (56.94)	562
Somalia	3 (10.34)	26 (89.66)	29
Tigray	291 (51.41)	275 (48.59)	566
**Year of reporting**			
2013	2 (15.38)	11 (84.62)	13
2014	129 (43.58)	167 (56.42)	296
2015	195 (39.71)	296 (60.29)	491
2016	379 (45.83)	448 (54.17)	827
2017	596 (45.81)	705 (54.19)	1301
2018	293 (42.96)	389 (57.04)	682
2019	225 (47.27)	251 (52.73)	476
2020	198 (44.59)	246 (55.41)	444

### Socio-demographic characteristics of the deceased women

Most of the women who died due to obstetric haemorrhage were within the age group between 40–49 years (63.11%) and the age between 10–19 years (49.19%). Women, who resided in rural areas, had a higher proportion of death due to obstetric haemorrhage (57.11%) than women who live in urban areas (46.58%). The majority of maternal deaths at home were due to obstetric haemorrhage (70.92%) in comparison to facility death (48.94%). The proportion of women who died as a result of obstetric haemorrhage was higher among women with a parity of more than five (67.22%) compared to those who had parity between zero and one (0–1) (46.17%) (Table 4).

**Table 4:** Percentage of obstetric haemorrhage by personal characteristics and medical history of the deceased women among reviewed maternal death in Ethiopia, 2020(N = 4530).

Characteristic	Deceased by obstetric hemorrhage		Overall, N = 4,530
	No, N = 2,017	Yes, N = 2,513	
**Age group**			
10_19Y	126 (50.81)	122 (49.19)	248
20_29Y	1,126 (49.02)	1,171 (50.98)	2,297
30_39Y	675 (38.77)	1,066 (61.23)	1,741
40-49Y	90 (36.89)	154 (63.11)	244
**Residence**			
Urban	375 (53.42)	327 (46.58)	702
Rural	1,642 (42.89)	2,186 (57.11)	3,828
**Place of death**			
Home	178 (29.08)	434 (70.92)	612
On transit	243 (30.68)	549 (69.32)	792
Health facility	1,596 (51.06)	1,530 (48.94)	3,126
**Marital Status**			
Unmarried	126 (42.86)	168 (57.14)	294
Married	1,891 (44.64)	2,345 (55.36)	4,236
**Religion**			
Traditional	12 (34.29)	23 (65.71)	35
Muslim	781 (46.19)	910 (53.81)	1,691
Christian	1,224 (43.65)	1,580 (56.35)	2,804
**Level of education**			
Secondary and above	224 (56.57)	172 (43.43)	396
Primary	232 (48.13)	250 (51.87)	482
Illiterate	1,561 (42.74)	2,091 (57.26)	3,652
**Parity category**			
0–1	850 (53.83)	729 (46.17)	1,579
2_4	789 (43.88)	1,009 (56.12)	1,798
≥5	378 (32.78)	775 (67.22)	1,153
**Time of death**			
Antepartum	50 (60.24)	33 (39.76)	83
Intrapartum	637 (51.45)	601 (48.55)	1,238
Post-partum	1,330 (41.45)	1,879 (58.55)	3,209
**History of ANC follow up**			
No	1,271 (42.11%)	1,747 (57.89%)	3,018
Yes	746 (49.34%)	766 (50.66%)	1,512

### Selected characteristics of maternal health

The proportion of women who died due to obstetric haemorrhage was higher among older aged (62.99%), grand multipara (66.37%), and those who had a history of uterine atony (91.57%). Women with limited access to the road had a higher proportion of death due to obstetric haemorrhage (68.9%) as compared to those who had access to the road (31.81%). Similarly, the proportion of women who died due to obstetric haemorrhage was much higher among women who did not have access to transportation (71.36%) than women with access to transportation (28.64%) (Table 5).

**Table 5:** Factors contributing to the adverse outcomes of obstetric hemorrhage among reviewed maternal death in Ethiopia, 2020 (N = 4530).

Characteristic	Deceased by obstetric hemorrhage		Overall, N = 4,530
	No (%), n = 2,017	Yes (%), n = 2,513	
**Medical factor**			
**Older age**			
Yes	438 (37.31)	736 (62.69)	1,174
No	1,579 (47.05)	1,777 (52.95)	3,356
**Grand multipara**			
Yes	531 (33.63)	1,048 (66.37)	1,579
No	1,486 (50.36)	1,465 (49.64)	2,951
**Being anemic**			
Yes	386 (47.65)	424 (52.35)	810
No	1,631 (43.84)	2,089 (56.16)	3,720
**Uterine atony**			
Yes	133 (8.43)	1,444 (91.57)	1,577
No	1,884 (63.80)	1,069 (36.20)	2,953
**Coagulopathy**			
Yes	712 (88.89)	89 (11.11)	801
No	1,305 (35.00)	2,424 (65.00)	3,729
**Injured during delivery**			
Yes	602 (47.07)	677 (52.93)	1,279
No	1,415 (43.53)	1,836 (56.47)	3,251
Non-medical factor			
**Delay one (decision to seek care)**			
**Family poverty**			
Yes	168 (51.38)	159 (48.62)	327
No	1,849 (43.99)	2,354 (56.01)	4,203
**Failed to decide to go to HF**			
Yes	590 (49.17)	610 (50.83)	1,200
No	1,427 (42.85)	1,903 (57.15)	3,330
**Traditional practice**			
Yes	299 (39.76)	453 (60.24)	752
No	1,718 (45.47)	2,060 (54.53)	3,778
**Having poor knowledge of obstetric complications**			
Yes	664 (41.50)	936 (58.50)	1,600
No	1,353 (46.18)	1,577 (53.82)	2,930
**The nearest HF is more than 5 km away**			
Yes	525 (40.35)	776 (59.65)	1,301
No	1,492 (46.21)	1,737 (53.79)	3,229
**Delay two (reaching care)**			
**Lack of road**			
Yes	111 (31.81)	238 (68.19)	349
No	1,906 (45.59)	2,275 (54.41)	4,181
**Lack of money for transport**			
Yes	63 (46.67)	72 (53.33)	135
No	1,954 (44.46)	2,441 (55.54)	4,395
**Lack of transportation**			
Yes	173 (28.64)	431 (71.36)	604
No	1,844 (46.97)	2,082 (53.03)	3,926
**Lack of healthcare facilities in the surrounding.**			
Yes	80 (39.41)	123 (60.59)	203
No	1,937 (44.77)	2,390 (55.23)	4,327
**Long travel time from home to a health facility**			
Yes	476 (44.03)	605 (55.97)	1,081
No	1,541 (44.68)	1,908 (55.32)	3,449
**Delay three (receiving care)**			
**Long travel time from HF to HF፟**			
Yes	410 (41.25)	584 (58.75)	994
No	1,607 (45.45)	1,929 (54.55)	3,536
**Scarcity of essential equipment and supplies**			
Yes	225 (47.47)	249 (52.53)	474
No	1,792 (44.18)	2,264 (55.82)	4,056
**Waiting for a longer duration before receiving treatment**			
Yes	253 (46.68)	289 (53.32)	542
No	1,764 (44.23)	2,224 (55.77)	3,988
**Mistaken during assessment, diagnosis, and treatment**			
Yes	151 (55.11)	123 (44.89)	274
No	1,866 (43.84)	2,390 (56.16)	4,256

### Latent class classification findings

A total of 20 adverse maternal health conditions were included in the latent class analysis that is composed of medical factors (older age, grand multiparity, being anemic, injury during delivery, diagnosis with uterine atony and coagulopathy) and non-medical factors, which were classified in three categories, namely: 1) decision to seek care (family poverty, traditional practice, nearest HF is more than 5 km away, lack of awareness on obstetric complications, failed to decide to go to health facility), 2) reaching care (lack of road, lack of transportation, lack of money for transport, lack of HF in the surrounding, extensive travel time from home to a HF) and 3) receiving care (long travel time from HF to HF፟, scarcity of essential equipment and supplies, waiting for a longer duration before receiving treatment, mistakes during assessment, diagnosis, and treatment).

**Fig 1:**
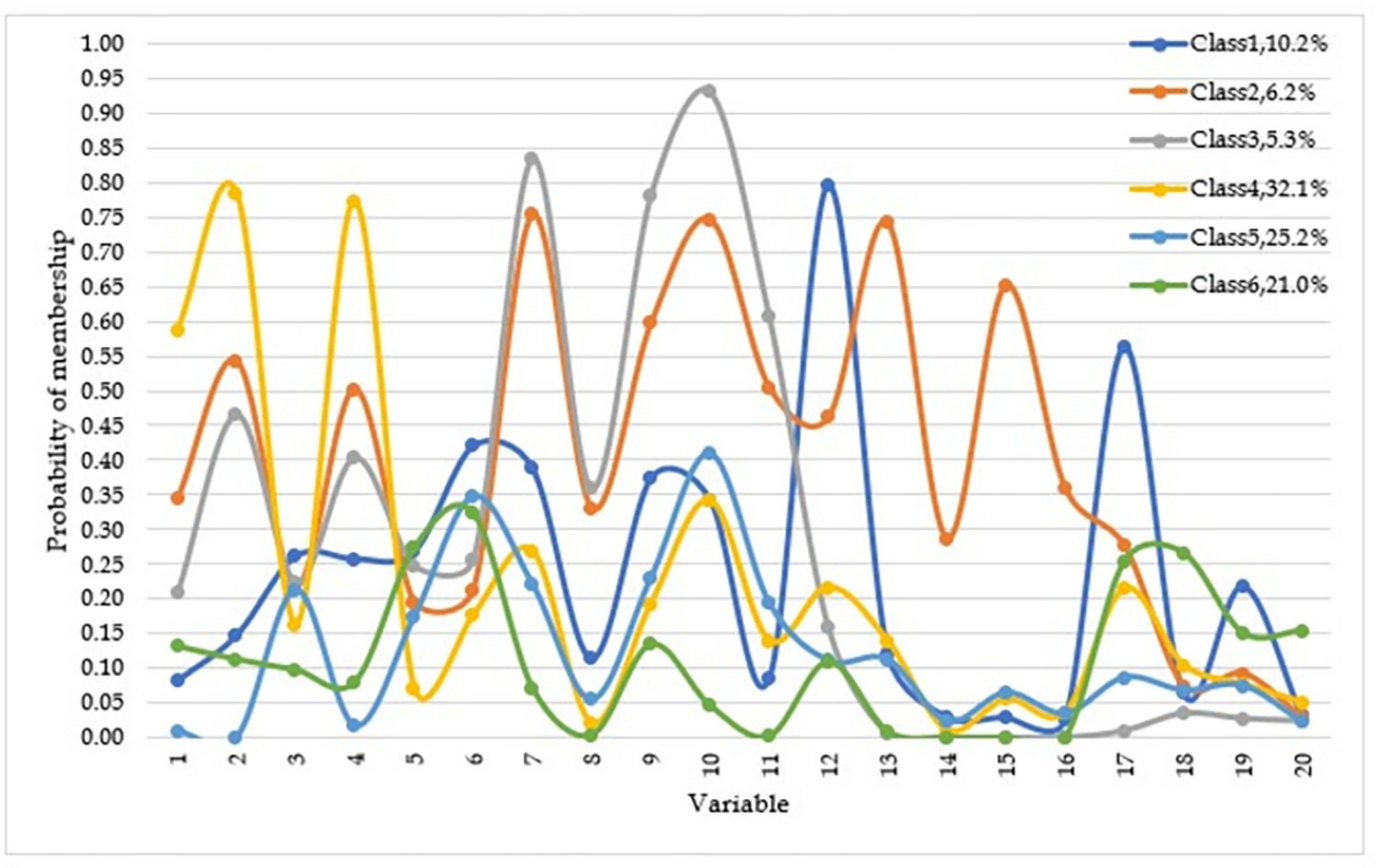
Latent classes of maternal health adversities increased adverse outcome of obstetric haemorrhage: Graphical displays of probabilities across each of the six classes 1) Older age, 2) Grand multipara, 3) Being Anaemic, 4) Uterine atony, 5) Coagulopathy, 6) Injured delivery, 7) Nearest HF is more than 5 km away, 8) Family poverty, 9) Lack of awareness of obstetric complications, 10) Failed to decide to go to a health facility, 11) Traditional practices 12) Long travel time from home to a healthcare facility, 13) Lack of transportation, 14) Lack of money for transport, 15) lack of road, 16) Lack of HF in the surrounding, 17) Long travel time from HF to HF, 18) Waiting for a longer duration before receiving treatment, 19) Scarcity of essential equipment and supplies, 20) Mistaken during an assessment, diagnosis, and treatment.

The LCA models were quantified and tested by using 2–6 classes. The best-fitted model, both statistically (based on the BIC) and theoretically was a six-class classification. The entropy value for the six-class model was 0.82, which indicates that there was a preferable precision in assigning mothers to the proper class. Table 6 shows the response probabilities of each adverse maternal health condition within six latent classes. Indicators with the highest average posterior probabilities within each class were selected for the nomenclature of the subgroup Moreover, the graphical presentation of each item’s response probabilities is displayed in Fig 1.

**Table 6:** Latent class analysis among reviewed maternal death in Ethiopia: Item-response probabilities and probabilities of latent class membership within the six classes, 2020 (N = 4530).

Variables	Class1_Long travel to reach care	Class2_ Lack of Access to HF	Class3_Failed to decide to go to HF	Class4_Grand Multipara	Class5_Injured during delivery with failed to decide to go to HF	Class6__ Injured during delivery with a history of coagulopathy
**Probability of latent class membership within classes**	(10.22%)	(6.15%)	(5.32%)	(32.09%)	(25.21%)	(21.02%)
**Item-response probabilities within each class**						
**Medical and personal factors**						
Older age	0.083	0.344	0.208	0.588	0.009	0.132
Grand multipara	0.148	0.543	0.465	0.784	0.000	0.113
Being anemic	0.262	0.219	0.224	0.163	0.213	0.098
Uterine atony	0.258	0.502	0.404	0.773	0.017	0.081
Coagulopathy	0.269	0.195	0.248	0.071	0.174	0.274
Injured during delivery	0.421	0.214	0.258	0.177	0.348	0.323
**Nonmedical factor**						
**Delay -1(decision to seek care)**						
Nearest HF is more than 5 km away	0.388	0.754	0.835	0.269	0.221	0.071
Family poverty	0.114	0.331	0.360	0.020	0.055	0.004
Having poor knowledge on obstetric complications	0.374	0.598	0.782	0.193	0.231	0.134
Failed to decide to go to health facility	0.343	0.748	0.931	0.341	0.410	0.046
Traditional practices	0.086	0.505	0.607	0.139	0.194	0.002
**Delay 2(reaching care)**						
Long travel time from home to a healthcare facility	0.797	0.462	0.158	0.217	0.111	0.109
Lack of transportation	0.122	0.743	0.006	0.139	0.113	0.009
Lack of money for transport	0.030	0.287	0.000	0.010	0.023	0.000
Poor road condition	0.030	0.652	0.000	0.055	0.065	0.000
Lack of HF in the surrounding	0.027	0.360	0.000	0.035	0.034	0.000
**Delay 3(receiving care)**						
Long travel time from HF to HF፟	0.564	0.277	0.010	0.215	0.087	0.255
Waiting for longer duration before receiving treatment	0.064	0.075	0.035	0.104	0.068	0.267
Scarcity of essential equipment and supplies	0.219	0.092	0.028	0.076	0.075	0.151
Mistaken during assessment, diagnosis, and treatment	0.031	0.029	0.024	0.050	0.023	0.154

Class 1 (Long travel time to reach HF) women in this class tend to travel for a longer duration to reach HF from home and to the next referral HF. This class contains over one-tenth (10.22%) of the entire sample.

Class 2 (Lack of Access to HF) women in this class have a greater probability of not accessing HF within a 5 km radius. Moreover, Women within this class were highly likely to fail to decide to go to the HF and had a greater probability of not obtaining transportation. This class constitutes 6.15% of the latent class.

Class 3 (failed to decide to go to HF) women in this latent class, were more likely to fail to decide to go to a health facility. They had no access to a facility within a reasonable distance and were unaware of obstetrics complications. This class contributes 5.32% of the sample

Class 4 (grand multipara) includes women with the highest probability of being grand multipara, older aged women, and having uterine atony. This is the largest class and represents almost one-third (32.09%) of the sample.

Class 5 (injured during delivery and failed to decide to go to HF) women in this latent class showed the highest probability of being injured during delivery and failed to decide to go to a facility for further treatment.

Class 6 (injured during delivery with a history of coagulopathy) women in this class had a relatively higher chance of being injured during delivery and were accompanied by coagulopathy. Moreover, women in this class have failed to receive treatment early after being admitted to a health facility. This class contributes (21.02%) to the sample.

### Associations between adverse maternal health conditions and obstetric haemorrhage

Class 6 (Injured during delivery with a history of coagulopathy) is a reference category. As depicted in Table 7, the final regression analysis demonstrated that women from two latent classes, grand multipara and having no access to health facilities within a 5km radius, had a high risk of dying due to adverse outcomes of obstetric haemorrhage. Among socio-demographic covariates, only the level of education had an association with obstetric hemorrhage (women with no education are 1.62 more likely to die due to obstetric haemorrhage than those educated up to secondary and above). Women with a history of ANC follow-up are 0.85 times less likely to die due to obstetric haemorrhage than those with no ANC follow-up. Moreover, the place of death is also positively related to death that occurred due to obstetric haemorrhage; women who died while on transit and at home are more likely to die because of obstetric haemorrhage, as compared to women who died in a health facility with an odd of 2.13 and 1.90 respectively. Besides, as the facilities’ level of care goes up, the probability of dying due to obstetric haemorrhage declines

**Table 7:** Regression analysis between possible risk factors and obstetric hemorrhage among reviewed maternal death in Ethiopia, 2020 (N = 4530).

Variable	Coefficient (S.E.)	AOR	95% CI
**Latent Class**			
Class1_Long travel time to reach care	0.27(0.12)	1.32*	(1.04,1.67)
Class2_ Lack of Access to HF	0.89(0.15)	2.43***	(1.82,3.25)
Class3_Failed to decide to go to HF	0.42(0.16)	1.53**	(1.12,2.08)
Class4_Grand multipara	1.54(0.09)	4.69***	(3.93,5.59)
Class5_Injured during delivery with failed to decide to go to HF	0.35(0.09)	1.42***	(1.18,1.69)
Class6_ Injured during delivery with a history of coagulopathy (rc)			
**Covariates**			
**Residence**			
Rural	0.12(0.10)	0.88	(0.72,1.08)
Urban(rc)			
**Educational level**			
Illiterate	0.48(0.11)	1.62***	(1.29,2.02)
Primary	0.17(0.14)	1.18	(0.89,1.56)
Secondary and above(rc)			
**History of ANC follow up**			
Yes	0.16(0.08)	0.85*	(0.74,0.99)
No (rc)			
**Regional class**			
Pastoralist region	0.04(0.22)	0.96	(0.63,1.46)
Agrarian region	0.26(0.17)	1.30	(0.93,1.81)
City administration (rc)			
**Type of facility**			
Tertiary HCL	0.41(0.10)	0.66***	(0.54,0.81)
Secondary HCL	0.20(0.09)	0.82*	(0.69,0.98)
Primary HCL (rc)			
**Place of death**			
On transit	0.75(0.10)	2.13***	(1.74,2.61)
Home	0.64(0.10)	1.90***	(1.57,2.29)
Health facility(rc)			

^a^ *P < 0.05

**P < 0.001

***P < 0.0001

^b^ HCL (health care level)

^c^ rc (reference category)

## Discussion

The study demonstrated that women who dwell in a rural area, with no education, with higher parity, and who delivered at home were more likely to die due to the adverse outcomes of obstetric hemorrhage. The study also revealed that adverse maternal health conditions often co-occur, a particular sub-group of women were liable to multiple maternal health adverse events. The study identified six subgroups of women that demonstrate distinct profiles of exposure to adverse maternal health conditions: 1) long travel time from home to HF, 2) lack of access to HF, 3) failure to decide to go to HF, 4) grand multipara, 5) injured during delivery, 6) failure to decide to go to HF and traumatized during delivery with coagulopathy. Women who traveled for a longer duration from home to HF were also likely to suffer due to the extended hour travel from HF to HF for further evaluation and treatment (long travel time to reach care), while women who had no access to HF within 5km radius were also unable to decide to go to HF and this might escalate the adverse outcome of obstetric hemorrhage. Women’s class of grand multipara were more likely to have uterine atony and cover the largest portion of the sample. Women who failed to decide to go to HF are characterized by poor access to HF and low-level awareness of obstetrics complications. For the remaining two classes, although both share the common characteristics of facing injury while delivery, the first class faces delay in management after admission, while the other class has a history of coagulopathy.

Additionally, the results demonstrated that more than half of the women (55.5%) were deceased due to the adverse outcome of obstetric hemorrhage. Among them, 41.5% died during the postpartum period. The prevalence of obstetric hemorrhage is much higher than the estimate in sub-Saharan Africa [45], whereas it is comparable with similar studies conducted in Ethiopia (Jimma) [46], Nigeria [47], and Tanzania [48]. The finding on death due to postpartum hemorrhage was comparable with the global estimate and it is also well aligned with the estimate in Burkina Faso, Philippines, India, and Indonesia [49]. The finding suggests that obstetric hemorrhage remains to be a prominent cause of maternal death in Ethiopia, and it requires a coordinated effort to reduce its burden.

Finally, the study demonstrated that women in the two classes (grand multipara and those who lack access to HF) have a stronger association with adverse outcomes of obstetric hemorrhage. Women in the class of grand multipara are characterized by advanced maternal age along with a history of uterine atony, which heightened the risk of dying due to obstetric hemorrhage [50, 51]. The finding was consistent with studies in Senegal, Mali [52], Zimbabwe [51], Ethiopia (Shire Endasselassie and Yirgalem) [53, 54], and Afghanistan [55]. This could potentially be due to the conditions that affect uterine contraction, such as blood clots and retained placenta or remnants of placenta tissue that could diminish the contractility of the uterine muscle. Moreover, advanced maternal age and multiparity have a multiplier effect on uterine contraction that could lead to profound bleeding and death. In addition, women in the class lacked access to HF, described by lack of access to a health facility within a 5 km radius, had low-level awareness of obstetric complications, failed to decide to go to HF, and had poor road access to visit a HF. Other studies have also shown that distance was the main barrier that discouraged women from the utilize maternity services, which may lead to adverse outcomes for maternal health [56–58]. This finding suggested that obstetric complications demand timely response, which is affected by distance

Per our assessment, the level of education of a woman has a significant positive association with the adverse outcomes of obstetric hemorrhage. Women with no education were more likely to die due to obstetric hemorrhage than women who attend school up to secondary and above. These results demonstrate that women’s education is an influential and modifiable determinant of maternal death in Ethiopia. The possible explanation for this relationship is the fact that less-educated women are likely to suffer from primary and secondary delays. It is usually connected with delays in deciding to seek care and reaching care. Furthermore, educated women can make informed decisions on the issues of their health and well-being [59, 60].

ANC follow-up is one of the commonest factors related to adverse maternal health outcomes in sub-Saharan Africa [61]. Women with ANC follow-up were less likely to die due to obstetric hemorrhage than women who did not attend ANC follow-up at least once. ANC visit has a vital role in identifying unanticipated complications related to pregnancy, and it is considered as a protective intervention for any pregnancy. As part of the ANC follow-up, the women’s risk assessment is periodically performed, and pregnant mothers are recommended to have institutional delivery. However, in Ethiopia, 57% of women attend below four ANC sessions, which is against the WHO recommendation of eight visits [62–64]. The possible barriers for not attending ANC visits and losing following up might be explained by the unavailability, and high cost, of transportation; poor service provision; lack of spousal support; and inadequate awareness about ANC services [65]. The finding recommends the need for an intensified effort to increase the coverage of ANC services, which could also serve as a precautionary measure to identify potential risk factors that result in an adverse outcome of obstetric hemorrhage.

The type of facility is one of the vital determinants of adverse outcomes of obstetric hemorrhage. The study indicated that as the level of care goes up, the risk of dying due to adverse outcomes of obstetric hemorrhage is reduced. In addition, the study revealed that three fourth of women who died due to the adverse outcomes of obstetric hemorrhage deceased during the postpartum period. This indicates that managing obstetric emergencies at a lower-level facility is a challenging endeavor. specifically, after the delivery of the fetus, which is the critical time to avert preventable maternal death, as it has a strong connection with the quality of maternity care [66]. The quality of maternity care may be compromised by the absence of trained personnel, infrastructure, strong referral, and quality emergency care practices [67–70]. Although the ambulance transportation system is in place, free of charge, to enhance the referral system in Ethiopia [71] there is still a long way to go in improving the referral system and the capacity at the lower-level facilities.

The study also demonstrated that the risk of dying due to the adverse outcomes of obstetric hemorrhage was lower at health facilities than at other places of death (home and on transit). The finding was coherent with studies conducted in Tanzania [72] and India [73]. One possible explanation would be the absence of life-saving assistance during referrals such as the Non-Pneumonic Anti-Shock Garment (NASG), which is one of the highly recommended lifesaving supplies for women with active maternal bleeding [74]. The other major cause of adverse outcomes of hemorrhage is home delivery. Women prefer home delivery due to a lack of ANC follow-up and the absence of a well-prepared birth plan [75]. Conversely, Ethiopia has launched an initiative focused on the construction of maternity waiting rooms (MWH) in health facilities as a means to enhance the accessibility of obstetric services and reduce maternal death due to secondary delay [76]. However, it was poorly utilized due to the long distance to reach the site, limited awareness, and acceptance by the community [77]. In general, this finding suggests that improving the utilization of NASG and MWH is crucial to reduce the adverse effects of hemorrhage and maternal mortality.

This study has several limitations. The first limitation is the fact that nearly all deaths were reported from public facilities only, and this could potentially bias the representativeness of the study. In addition, all notified maternal deaths, identified through a weekly report, were not investigated and reported via MDFR to the next level, and this could also affect the generality of the finding. Moreover, the Latent class analysis is influenced by the number of indicators, and this study may not comprise all the adverse maternal health exposures that led to the unwanted outcome of obstetric hemorrhage.

## Conclusion

This study is one of the pioneer assessments conducted to explore maternal health adverse exposure and its association with obstetric hemorrhage in Ethiopia. The findings of this assessment shed light on a range of exclusive adverse maternal health exposures faced by mothers living in a lower resource setting. The study findings suggest that women with personal factors (multipara, history of uterine atony, and older age) and contributing factors (lack of access to a facility within a reasonable distance, poor road condition, transposition, and knowledge of obstetrics complications) are more likely to be affected due to the adverse outcome of obstetric hemorrhage. The study provides initial evidence for targeted measures to reduce maternal death due to obstetric hemorrhage. Local, provincial, and national stakeholders are expected to conduct further assessments to introduce an effective maternal health intervention in achieving the aspired goals under the sustainable development goal.
